# Comprehensive Essentiality Analysis of the *Mycobacterium tuberculosis* Genome via Saturating Transposon Mutagenesis

**DOI:** 10.1128/mBio.02133-16

**Published:** 2017-01-17

**Authors:** Michael A. DeJesus, Elias R. Gerrick, Weizhen Xu, Sae Woong Park, Jarukit E. Long, Cara C. Boutte, Eric J. Rubin, Dirk Schnappinger, Sabine Ehrt, Sarah M. Fortune, Christopher M. Sassetti, Thomas R. Ioerger

**Affiliations:** a Department of Computer Science and Engineering, Texas A&M University, College Station, Texas, USA; b Department of Immunology and Infectious Diseases, Harvard TH Chan School of Public Health, Boston, Massachusetts, USA; c Department of Microbiology and Immunology, Weill Cornell Medical College, New York, New York, USA; d Department of Microbiology and Physiological Systems, University of Massachusetts Medical School, Worcester, Massachusetts, USA; e Howard Hughes Medical Institute, Chevy Chase, Maryland, USA; Washington University in St. Louis School of Medicine

## Abstract

For decades, identifying the regions of a bacterial chromosome that are necessary for viability has relied on mapping integration sites in libraries of random transposon mutants to find loci that are unable to sustain insertion. To date, these studies have analyzed subsaturated libraries, necessitating the application of statistical methods to estimate the likelihood that a gap in transposon coverage is the result of biological selection and not the stochasticity of insertion. As a result, the essentiality of many genomic features, particularly small ones, could not be reliably assessed. We sought to overcome this limitation by creating a completely saturated transposon library in *Mycobacterium tuberculosis*. In assessing the composition of this highly saturated library by deep sequencing, we discovered that a previously unknown sequence bias of the *Himar1* element rendered approximately 9% of potential TA dinucleotide insertion sites less permissible for insertion. We used a hidden Markov model of essentiality that accounted for this unanticipated bias, allowing us to confidently evaluate the essentiality of features that contained as few as 2 TA sites, including open reading frames (ORF), experimentally identified noncoding RNAs, methylation sites, and promoters. In addition, several essential regions that did not correspond to known features were identified, suggesting uncharacterized functions that are necessary for growth. This work provides an authoritative catalog of essential regions of the *M. tuberculosis* genome and a statistical framework for applying saturating mutagenesis to other bacteria.

## IMPORTANCE

Sequencing of transposon-insertion mutant libraries has become a widely used tool for probing the functions of genes under various conditions. The *Himar1* transposon is generally believed to insert with equal probabilities at all TA dinucleotides, and therefore its absence in a mutant library is taken to indicate biological selection against the corresponding mutant. Through sequencing of a saturated Himar1 library, we found evidence that TA dinucleotides are not equally permissive for insertion. The insertion bias was observed in multiple prokaryotes and influences the statistical interpretation of transposon insertion (TnSeq) data and characterization of essential genomic regions. Using these insights, we analyzed a fully saturated TnSeq library for *M. tuberculosis*, enabling us to generate a comprehensive catalog of *in vitro* essentiality, including ORFs smaller than those found in any previous study, small (noncoding) RNAs (sRNAs), promoters, and other genomic features.

## INTRODUCTION

Deep sequencing of transposon (Tn) insertion (TnSeq) libraries has become a powerful tool for evaluating the essentiality of genomic features in bacterial organisms. Random Tn insertions can disrupt the functions of genes and regulatory regions. The consequent effects on growth can be efficiently quantified by amplifying and sequencing the transposon-chromosome junctions from a complex library, a technique known as TnSeq (1–3). Comparative analysis of genes that are essential under different environmental conditions has been used to dissect specific metabolic pathways (4) and mechanisms of pathogenesis (5, 6). Similarly, comparing the effects of Tn insertion in different genetic backgrounds can reveal genetic interactions that imply functional relationships between genes (7–9).

Comparative analyses to identify conditionally essential genes are fairly robust because they require only the accurate estimation of each mutant’s relative abundance in two libraries (10–12). In contrast, identifying the primary functions necessary for growth even under favorable *in vitro* conditions represents a greater statistical challenge. These analyses rely on the characterization of a single transposon library to identify regions that are devoid of transposon insertions and therefore likely to encode functions that are necessary for growth (6, 13). Many of these procedures rely on the *Himar1* transposon, which is used because it is thought to lack sequence specificity except for the required TA dinucleotide insertion site (14). Previous analyses of *Himar1* libraries of *Mycobacterium tuberculosis* suggest that there are approximately 600 genes that are essential for growth of this bacterium in standard laboratory medium (4, 15). This number represents approximately 15% of this organism’s genomic content, which is consistent with estimates from studies of other bacteria with similarly sized genomes (16). The identified functions include many well-known housekeeping genes involved in DNA replication, protein translation, cell growth and division, and core metabolic pathways. There are several widely used reference TnSeq data sets for *M. tuberculosis*, including those of Griffin et al. (4) and Zhang et al. (6). The essential genes identified in these studies are broadly consistent with the original hybridization-based technique (known as “TraSH” [15]). However, there are differences between the studies in the predicted essentiality of some genes. Some of these discrepancies may reflect differences in both growth conditions and methodology, such as sequencing depth (number of reads) and integration of barcodes to reduce the effect of PCR bias (17).

A less-appreciated limitation of the published TnSeq studies of *M. tuberculosis* is that they relied on libraries that were saturated only moderately (50% to 60%). In this situation, there is a relatively high probability that a nonessential region would be devoid of insertions due to chance rather than selection. As a result, the published TnSeq analyses had difficulty confidently assessing the essentiality of small genes with only a few TA sites. For instance, no genes with fewer than 6 TA sites were classified as essential by Griffin et al. (4) or Zhang et al. (6), despite the fact that there are 538 small open reading frames (ORFs) with 1 to 5 TA sites in the *M. tuberculosis* genome. Hence, ~13% of the ORFs of *M. tuberculosis* (538 of 3,990 genes) were effectively excluded from TnSeq profiling in those prior studies.

Low saturation levels have, to some extent, limited the statistical power of all previous attempts to define essential regions of a bacterial genome. As the *M. tuberculosis* genome contains only 74,602 TA insertion sites, and as millions of random mutants can be generated, we sought to overcome the uncertainty inherent in previous studies by saturating the possible *Himar1* insertion sites. In this work, we report a large-scale analysis of 14 independent TnSeq libraries in *M. tuberculosis* H37Rv, representing a combined total of 35,314,576 independent insertion events. All libraries were treated uniformly, grown in standard laboratory medium, and sequenced to a great depth using molecular barcodes. This unprecedented level of saturation enabled us to analyze sequence preferences for insertion, which revealed an unexpected sequence motif that is less permissive for Tn insertion. We used a hidden Markov model (HMM) which accounts for the reduced insertability of TA sites matching this motif to identify essential genes and genomic regions. This allowed us to assess the essentiality of small genomic regions, including small ORFs, promoters, and small (noncoding) RNAs (sRNAs). The analysis of a saturated *Himar1* library provides a more reliable and comprehensive catalog of essential genomic features in *M. tuberculosis* and provides the basis for similar studies in other bacteria.

## RESULTS

Generating a definitive map of essential genomic features in *M. tuberculosis* required the ability to interrogate small loci that contained few TA insertion sites. We reasoned that our ability to assess the essentiality of small features would be enhanced by producing a fully saturated library of mutants. To do this, we generated fourteen independent Tn insertion libraries in the H37Rv strain of *M. tuberculosis* by transfection with the ΦMycoMarT7 vector carrying the *Himar1* transposon (15, 17). In total, these libraries contained 35,314,576 independent insertion events, representing a level of coverage of 84.3% of the potential TA insertion sites. The transposon-chromosome junctions in each library were amplified and sequenced as previously described (15, 17). Sequencing the 14 libraries yielded an average of 2.5 million unique transposon-chromsosome junctions (termed “template counts”), which could be mapped to 42% to 64% of the TA dinucleotide sites in the chromosome in each individual library (see Table S1 for sequencing statistics).

10.1128/mBio.02133-16.4 TABLE S1  Sequencing statistics for the 14 replicate data sets. Tw, Tween-80; Kan, kanamycin. Download TABLE S1, DOCX file, 0.01 MB. Copyright © 2017 DeJesus et al. 2017 DeJesus et al. This content is distributed under the terms of the Creative Commons Attribution 4.0 International license.

### Saturating mutagenesis of fitness-neutral sites can be achieved.

**FIG 1:**
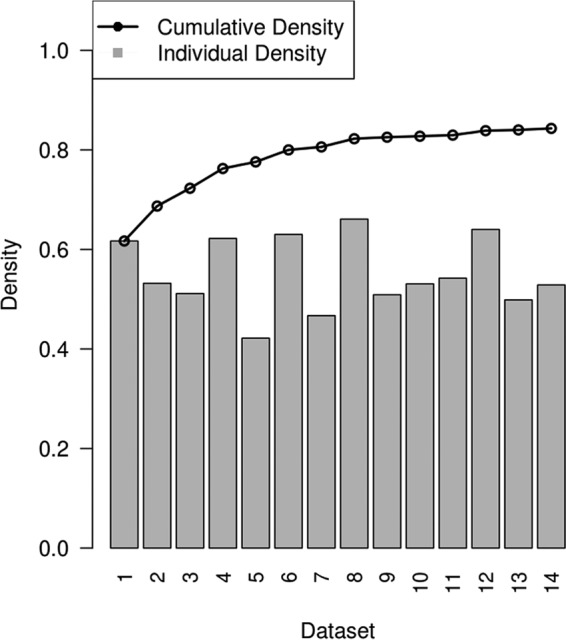
Cumulative fraction of TA sites represented as independent TnSeq data sets (black line). The gray bars show the saturation level of the individual data sets.

To assess the level of saturation in the aggregated data set, we determined the cumulative density of insertions as each of the 14 libraries was included (in random order). Insertion density reached a plateau after about 12 of the 14 data sets were included (Fig. 1), indicating that virtually all available fitness-neutral sites contained an insertion in at least one library. In the aggregated data set, a *Himar1* insertion was detected in 84.3% of the TA sites, indicating that insertional mutants corresponding to 11,712 TA sites (15.7%) could not be recovered under these conditions. Many of the unoccupied sites clustered in regions of more than four consecutive TAs, and most of these gaps corresponded to ORFs that are essential for growth. When these operationally defined essential regions are excluded, the overall saturation increases to 92.5% (Table 1). The fact that 7.5% of the remaining TAs were unoccupied indicates the presence of many smaller essential genomic features that contained 1 to 4 TAs that did not sustain insertions in any of the libraries. These unoccupied sites were not generally correlated with sequencing failures (i.e., did not correspond to poorly sequenced regions of the genome in other *M. tuberculosis* samples resequenced on the Illumina platform). However, it remained possible that some of these unoccupied sites corresponded to regions that were underrepresented as a result of either a moderate fitness defect in the corresponding mutant or previously uncharacterized insertion preferences of *Himar1* that are detectable only in a library as highly saturated as this. As described below, both of these effects contributed to the observed unoccupied sites and need to be taken into account.

**TABLE 1:** Insertion count statistics of TA sites

TA site category	No. (%) of sites	% saturation	Mean read count (nonzero sites)
All	74,602	84.3	182.27
Those in putative nonessential regions^a^	67,992	92.5	182.27
Those in high-coverage regions^b^	57,452	96.5	189.54
Those in HC regions not matching NP sequence motif	52,672	98.8	192.18
All matching the NP motif	6,659 (9%)	59.7	50.00
All not matching the NP motif	67,943 (91%)	96.1	184.94

a Nonessential regions are defined as regions not containing a run of 4 or more unoccupied sites.

b High-coverage regions are based on labeling by the segmentation algorithm.

### Filtering out low-coverage regions reduces the fraction of unoccupied sites.

We noted that many of the unoccupied sites were found in regions with lower-than-average insertion counts. Furthermore, the adjacent TA sites with low counts were often represented in only a small subset of the 14 independent libraries. If the representation of insertions in these libraries were stochastic, the number of independent libraries with an insertion at each biologically neutral site would be expected to be distributed binomially (sum of 14 Bernoulli trials). However, the observed distribution was significantly different from would be expected by chance (see Fig. S1a in the supplemental material). For example, sites with insertions in fewer than 3 of 14 replicates would be expected to be extremely rare if representation were completely random, and yet TA sites in putatively nonessential regions with insertions in just a few of the libraries (1–3) are frequently observed in the data. Template counts observed at each TA site exhibited a rough correlation with the number of replicates in which an insertion was detected (Fig. S1b), indicating that the abundance of a mutant is correlated with the probability that it would be detected. Note that the distributions shown in Fig. S1 exclude obvious “hit-free” essential regions and show that the insertions are still not completely random in the remaining nonessential regions but that there are various degrees of essentiality. These “low-coverage” (LC) regions likely correspond to genes that produce a quantitative growth defect upon disruption (15, 18), leading to underrepresentation in the libraries. Since detection of insertions in these low-coverage regions is less reliable, we sought to eliminate them from the analysis.

10.1128/mBio.02133-16.1 FIG S1  (a) Distribution of the numbers of replicates with insertions (*x* axis) (blue bars) compared to those calculated on the basis of simulating insertions as Bernoulli trials (red bars) for TA sites in putative nonessential regions. (b) Box plot showing that sites with insertions in fewer replicates have lower mean read counts. Download FIG S1, TIF file, 8.1 MB. Copyright © 2017 DeJesus et al. 2017 DeJesus et al. This content is distributed under the terms of the Creative Commons Attribution 4.0 International license.

To systematically filter out TA sites in low-coverage regions, we implemented a probabilistic method for segmenting the genome into high-coverage (HC) and low-coverage (LC) regions (see Text S1 in the supplemental material). The segmentation algorithm effectively clusters TA sites together based on the local magnitude of read counts. The segmentation algorithm identified 833 LC regions, spanning 23% of the TA sites in the genome. This included all of the putative essential regions based on the operational definition given above (containing runs of 5 or more consecutive TA sites with no insertions), as well as isolated sites with 0 insertions adjacent to sites with depressed counts. Importantly, there were only 2,007 unoccupied TA sites remaining among the 57,452 TA sites in high-coverage regions (96.5% saturation). Of these remaining unoccupied sites, 86% were isolated TA sites surrounded by sites with insertions on both sides, and 12% were adjacent pairs of unoccupied sites.

10.1128/mBio.02133-16.10 TEXT S1  Segmentation algorithm for identifying low-coverage regions. Download TEXT S1, PDF file, 0.1 MB. Copyright © 2017 DeJesus et al. 2017 DeJesus et al. This content is distributed under the terms of the Creative Commons Attribution 4.0 International license.

### Some TA sites appear to be nonpermissive for *Himar1* insertion.

**FIG 2:**
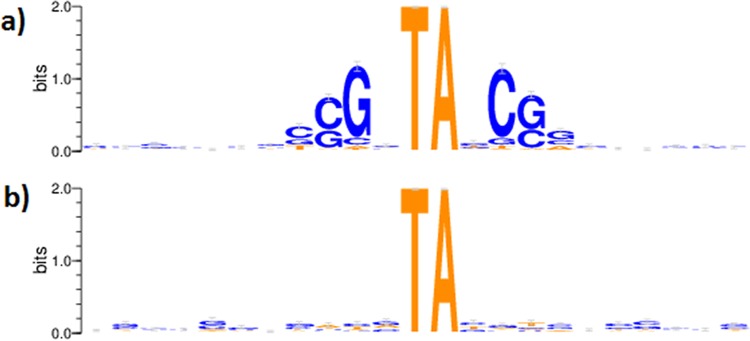
(a) Logo plot of log_2_ of nucleotide frequencies surrounding TA sites in the set of 1,746 unoccupied sites found in high-coverage regions (nonpermissive set). (b) Logo plot of log_2_ of nucleotide frequencies surrounding TA sites in the permissive set.

We then sought to determine if any of the remaining 2,007 unoccupied TA sites in HC regions might reflect a sequence preference for *Himar1* Tn insertion making them “nonpermissive” (NP) for insertion. To look for a sequence pattern associated with such sites, we selected a putatively nonpermissive (NP) set of 1,746 unoccupied TA sites in high-coverage regions not associated with known genomic features other than ORFs (such as tRNAs, rRNAs, promoters, DNA methylation sites, etc., which could be devoid of insertions due to biological selection; see Materials and Methods). A corresponding set of 1,746 “permissive” sites (those with the highest mean template counts, >554) was chosen for comparison. The logo plot of the nucleotides surrounding TA sites in the nonpermissive set (Fig. 2a) reveals a strong sequence preference. Specifically, almost all of the nonpermissive sites have G at −2 and C at +2 around the TA dinucleotide, and sites at ±3 are either G or C. Forty-four percent of nonpermissive sites matching this pattern were nonpalindromic. Thus, the sequence pattern (GC)GNTANC(GC) is strongly associated with nonpermissiveness. In contrast, the permissive sites exhibit no local sequence motif that could be interpreted as favoring insertion (Fig. 2b).

**FIG 3:**
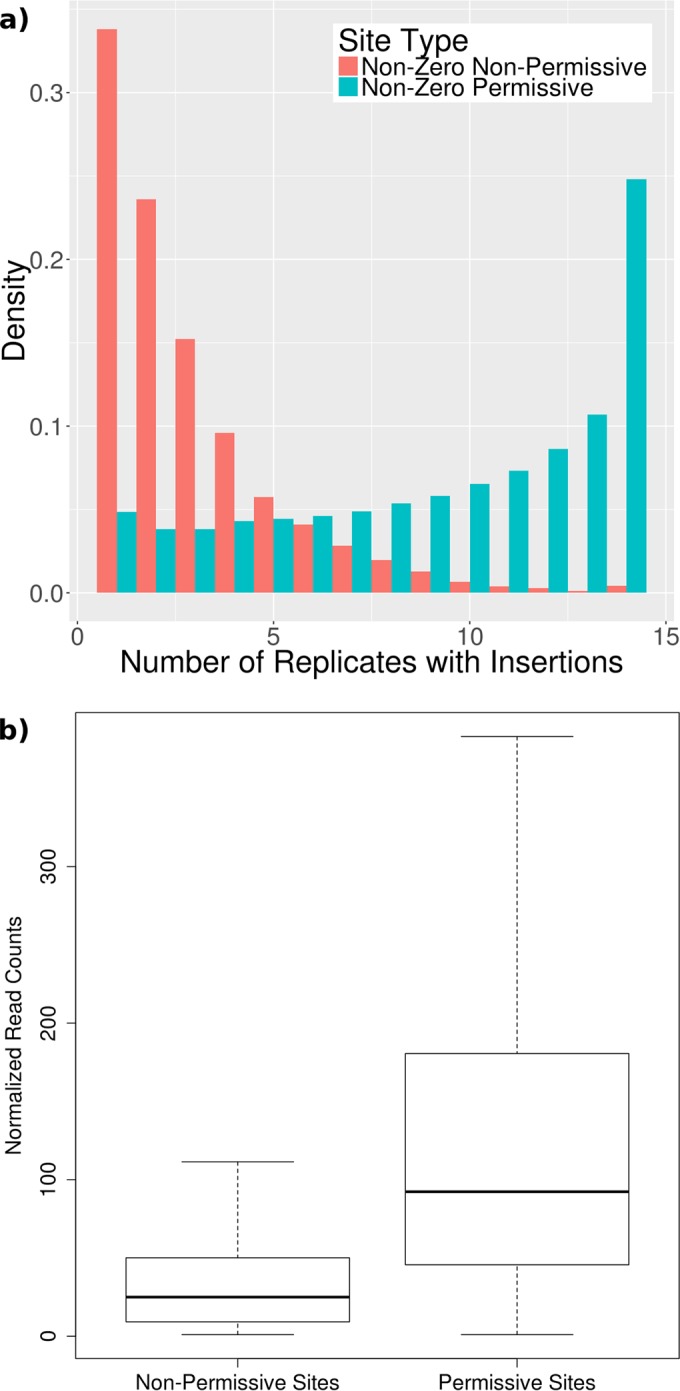
(a) Number of libraries representing sites matching (GC)GNTANC(GC) occupied by at least 1 insertion (red), compared to distribution over all TA sites (blue). (b) Box plot of nonzero insertion counts at sites matching the NP sequence motif versus sites not matching the motif. The boxes show the 25% to 75% interquartile range, while the whiskers show the majority of the range of insertion counts, except for the most extreme outliers.

Two-thirds of the TA sites in the nonpermissive set (1,223/1,746) match the sequence pattern (GC)GNTANC(GC), while none of the 1,746 sites in the permissive set match this pattern. The remaining sites not covered by this pattern could lack insertions because they are similar to the pattern but do not perfectly match the consensus, or for other reasons, including biological selection. Over the whole genome, 6,659 of 74,602 sites (9%) match the nonpermissive sequence pattern. Forty percent of these (2,682) have no insertions in any of the 14 replicates, and of those with insertions, 82% had insertions in 4 or fewer libraries (Fig. 3a). The insertion counts at these sites were also generally suppressed (Fig. 3b). Table S2 in the supplemental material indicates whether each TA site in the genome matches the nonpermissive sequence pattern, along with the observed insertion counts for each site.

10.1128/mBio.02133-16.5 TABLE S2  Spreadsheet showing insertion data for each TA site. (A) Coordinate in H37Rv (NC_018143.1). (B) Indicator of whether the site matches the nonpermissive sequence motif. (C) Number of the replicates with insertions. (D) Sum of normalized read counts across 14 replicates. (E) Means of normalized read counts among replicates with insertions. (F) Indicator of whether site is in a low-coverage region based on HMM. (G) Identifier (ID) of ORF spanning the site. (H to M) Other annotations of regions, including tRNAs, rRNAs, DNA methylation sites, predicted rho-independent termination signals, 5′UTRs, and promoters (as described in Materials and Methods). Most of these regions are indicated as a consecutive sequence of TA sites sharing a numbered identifier, such as promoter PROM-1925 (4 TA sites within a window of −150 to +70 bp around the transcriptional start site for Rv0161). (N) Essentiality state assignment from the HMM of essentiality. Download TABLE S2, XLSX file, 3.1 MB. Copyright © 2017 DeJesus et al. 2017 DeJesus et al. This content is distributed under the terms of the Creative Commons Attribution 4.0 International license.

After filtering out TA sites matching the nonpermissive (NP) sequence pattern, only 1.2% of the TA sites in high-coverage regions were devoid of insertions. Due to the saturation of the library, the odds that these sites are unoccupied due to chance are quite low. More likely, they are unoccupied because of unappreciated restrictions on Tn insertion or biological selection (i.e., disruption of a small essential genomic feature, the generation of a toxic truncated protein, etc.). For instance, Rv3397c contains an isolated but permissive site without insertions that coincides with a DNA methylation site, which could explain the apparent essentiality of this site. However, we cannot exclude the possibility that isolated sites in general are unoccupied because of biases against Tn insertion *per se*, making the interpretation of isolated unoccupied sites ambiguous. Thus, we are hesitant to conclude that isolated unoccupied TA sites necessarily correspond to a feature that is essential for growth. On the other hand, if we assume that unoccupied sites in nonessential regions are randomly distributed throughout the chromosome, the probability of two unoccupied (but permissive) sites being adjacent is <0.0001. Hence, we restrict the analysis of essentiality to regions with two or more TA sites (separated by at least 15 bp, to ensure independence).

### Nonpermissiveness for *Himar1* insertion is also observed in other prokaryotes.

TA sites matching the NP sequence motif are underrepresented in other bacteria as well, suggesting that this pattern might reflect a general disinclination for insertion by the *Himar1* transposon (see Table 2). For example, in a Tn library of *Haemophilus influenzae* (1), the overall saturation was 53%, whereas only 6% of those sites matching the NP pattern were occupied by insertions. Similarly, in a high-saturation *Himar1* library of *Caulobacter crescentus* with 85.3% insertion density overall (19), 42.7% of TA sites matching the NP motif had insertions (and 20% of these were represented by only single reads). In a *Himar1* transposon-insertion library in *Vibrio cholerae* (20), insertions were observed in 56% of the TA sites, but only 7% of those matching the NP motif had insertions. The generality of this bias against insertions at such sites in organisms beyond mycobacteria suggests that it might reflect an intrinsic property of the *Himar1* transposase, which has been noted to require severe distortion (bending) of the DNA at the insertion site (21).

**TABLE 2:** Statistics for TnSeq data sets analyzed to determine transposon bias at sites matching NP motif

Organism	Tn	Study	%GC	No. of TA sites	No. of NP sites	% density	% density at NP sites
*Mycobacterium tuberculosis*	*Himar1*	This study	66	74,602	6,659	84	60
*Caulobacter crescentus*	*Himar1*	Murray et al. (19)	67	44,708	1,672	94	43
*Haemophilus influenzae*	*Himar1*	Gawronski et al. (1)	38	131,954	814	53	6
*Vibrio cholerae*	*Himar1*	Chao et al. (20)	47	192,681	4,439	56	7
*Desulfovibrio vulgaris*	Tn*5*	Fels et al. (69)	63	61,769	2,687	7^a^	8
*Methanococcus maripaludis*	Tn*5*	Sarmiento et al. (24)	33	133,503	514	5	6
*Salmonella enterica* serovar Typhi	Tn*5*	Langridge et al. (23)	53	233,259	9,289	3	4
*Rhodopseudomonas palustris*	Tn*5*	Pechter et al. (70)	65	72,385	3,844	3	3

a Although the Tn*5* transposon can insert at many different sites, the analysis was restricted to TA dinucleotides to investigate if Tn*5* had difficulty inserting in sites matching the NP motif as well.

**FIG 4:**
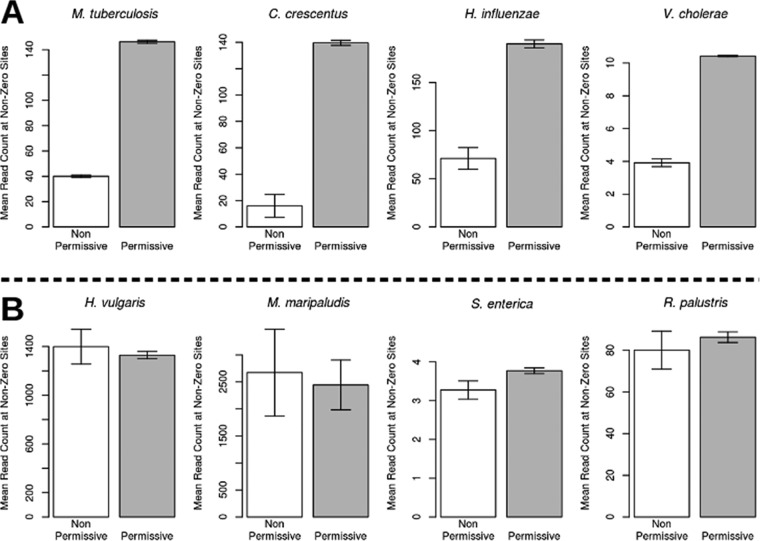
Mean read count at sites with at least one insertion for data sets made with the *Himar1* transposon (A) and the Tn*5* transposon (B). The nonpermissive sites (white bar), which match the nonpermissive motif identified in this study, significantly suppressed the read counts relative to sites that do not match the motif (permissive sites; grey bar). In contrast, limiting the analysis of the Tn*5* data sets to only insertions at TA dinucleotides, the mean read counts are similar for the permissive and nonpermissive sites. Error bars show the standard errors of the means.

To discount the possibility that there might be a physical factor prohibiting transposon insertion at such sites, such as blocking by a widely distributed DNA-binding protein or a peculiar distortion of the DNA double helix by the adjacent G and C nucleotides, we examined several data sets from libraries generated with the Tn*5* transposon, which comes from a completely different family of transposases. Tn*5* can insert at a wide variety of genomic locations, due to a weaker sequence preference bias (22), and is not just restricted to TA sites, though some TA sites are candidate insertion sites for Tn*5*. If attention is restricted to just TA sites, then there does not appear to be a suppression of transposon insertions at the subset of TA sites matching the NP motif (Table 2). For example, in a Tn*5* library of *Salmonella enterica* serovar Typhi (23), the frequency of insertions at all TA sites was 3.0%, while the density among TA sites matching the NP motif was 3.9%. Similarly, 4.6% of all TA sites in a Tn*5* library of *Methanococcus maripaludis* (24) were occupied, whereas 5.6% of TA sites matching the NP motif were represented. This pattern is also observed for the magnitude of read counts. Sites matching the NP motif had significantly reduced read counts in the *Himar1* data sets compared to other sites (see Fig. 4). Together, these observations suggest that the suppression of insertions reported here represents a previously unidentified local sequence bias of the *Himar1* transposase.

### Analysis of essential ORFs in H37Rv.

The aggregated data set was analyzed using a HMM similar to one described in reference 25, extended to take the nonpermissive motif into account. The HMM is a statistical model for sequential data that uses likelihood functions and observed insertion counts to infer which essentiality state is most likely at each TA site, but also integrates this information with neighboring sites to produce a locally consistent (smoothed) interpretation of essentiality across the genome. The HMM parses the genome into distinct regions in a non-gene-centered way, unbiased by annotated ORF boundaries. Briefly, read counts are modeled as coming from geometric distributions conditioned on four different states of essentiality: essential (ES), growth defect (GD), nonessential (NE), and growth advantage (GA). Parameters for the expected read count distributions for each state were set relative to the mean read count (with ES being near 0, NE being near the mean, GD approximately 1/10 the mean, and GA 5 times the mean). The likelihood parameters for sites matching the nonpermissive motif were scaled down empirically to account for the suppression in read counts observed at these sites (see Materials and Methods).

The extended HMM was applied to the aggregated library and was used to determine the most likely sequence of essentiality states for TA sites across the entire H37Rv genome, resulting in 11.6% of TA sites labeled ES, 77% NE, 3.5% GD, and 7.9% GA. The inferred state sequence was used to classify individual ORFs based on the most frequent label among TA sites in the ORF (the classification criteria are described in Materials and Methods). A total of 461 genes were identified as being essential and 135 genes with suppressed counts, whose disruption produces an apparent growth defect. As the HMM effectively recognizes large gaps (runs of consecutive TA sites lacking insertions) in ORFs, it is tolerant of a few insertions at the N and C termini of a gene. In addition, a subset (*n* = 29) are classified as “domain essentials,” as they have both significant essential and nonessential regions. Combined, these represent 625 genes which are necessary for optimal growth *in vitro* (see Table S3). This number is similar to the overall number of essentials (614) in H37Rv determined by traditional hybridization-based methods (15) and includes many well-known genes known to be necessary for essential cellular functions. Unlike previous analyses performed with subsaturated libraries, which classified several members of the MmpL family of integral membrane proteins as essential (4), the current analysis predicts that only MmpL3 is essential whereas the other 12 MmpL family members are dispensable *in vitro*. This is consistent with genetic deletion studies (26, 27), as well as with recent reports of MmpL3 as a potential drug target (28–30). Similarly, none of the 61 Pro-Glu (PE) or Pro-Pro-Glu (PPE) (PE_PGRS) genes are classified as essential, and almost all of the 67 PPE and 33 PE genes are classified as nonessential, as expected, given that these highly duplicated gene families do not appear to play a critical biological role *in vitro* (31). Of these gene families, only Rv0285/PE5 and Rv0286/PPE4 (part of the ESX-3 locus, which plays an essential role in iron acquisition [32]) are categorized as essential in this analysis. Note that PE_PGRS genes, which are GC rich and sometimes difficult to sequence, were enriched for nonpermissive sites; 35.6% of TA sites in PE_PGRS genes matched the NP pattern, compared to 9% overall. The inability to account for these insertional preferences is likely responsible for the inaccurate classification of some of these genes in previous studies. The gene encoding ICL (isocitrate lyase), which is a member of the glyoxylate shunt and is required for metabolizing fatty acids as a carbon source, was among the GD genes identified (33, 34). Because *M. tuberculosis* does not utilize carbon catabolite repression (34), cells with intact ICL grow better than ICL mutants on media containing fatty acids (e.g., oleate), but they can still survive without ICL, given the other carbon sources available in the rich medium used for culturing the libraries, explaining the growth defect phenotype.

10.1128/mBio.02133-16.6 TABLE S3  Spreadsheet of essentiality calls using the motif-dependent hidden Markov model of essentiality for all ORFs in the *M. tuberculosis* H37Rv genome. Column M contains the final essentiality call for the ORF as follows: essential (ES), essential domain (ESD), growth defect (GD), nonessential (NE), growth advantage (GA), and uncertain (for short empty genes). See Materials and Methods for the criteria used to classification of genes on the basis of the sequence of essentiality states obtained from the HMM. Download TABLE S3, XLSX file, 0.3 MB. Copyright © 2017 DeJesus et al. 2017 DeJesus et al. This content is distributed under the terms of the Creative Commons Attribution 4.0 International license.

Of the remaining genes, 3,008 were classified as NE by the HMM and as dispensable for growth *in vitro*, and 310 were classified as GA, indicating that disruption of these genes provides a growth advantage *in vitro*.

To compare these predictions to those of previous TnSeq studies, we reanalyzed data derived from a subsaturated library of H37Rv grown *in vitro* on glycerol as a carbon source (4) after applying a consistent multiple-testing correction and adjusted *P* value cutoff (*P*_adj_ ≤ 0.05). We found that 79% (456) of the genes predicted to be essential in the prior study were classified as essential or growth defect genes using the saturated library. Many of the classifications of the genes that differ with respect to essentiality, such as *pckA* (encoding phosphoenolpyruvate carboxykinase), *glpK* (encoding glycerol kinase), and *bioABDF1* (biotin biosynthesis genes), can be explained by differences in growth medium. While Griffin used minimal media plus glycerol, our replicates were grown on rich media (7H9 or 7H10 [7H9/10] plus oleic acid-albumin-dextrose-catalase [OADC]) with a variety of potential carbon sources available (glycerol, dextrose, oleate, citrate, and glutamate). In addition, we suspect that a number of the genes were misclassified as essential in previous studies due to the incomplete saturation of the libraries.

### Saturation enhances sensitivity for detecting small essential ORFs.

The high saturation of our data set enabled us to identify small ORFs that are essential for growth. Typically, TnSeq analysis methods have been limited in their ability to detect genes with fewer than 9 TA sites (4, 6), even though these represent a larger number of ORFs in the *M. tuberculosis* genome (i.e., 1,274 ORFs with 2 to 9 TA sites). The difficulty in analyzing small ORFs is primarily due to the subsaturation of the TnSeq libraries, as well as limitations inherent in the choice of statistical method.

Using the motif-dependent HMM on the saturated libraries, we found 92 of 1,274 small ORFs (7.2%) to be essential *in vitro*, which is comparable to the fraction of larger genes that are classified as essential (Fig. S2). We identified several small essential genes with well-known functions important for survival, including *ssb* (encoding a single-stranded binding protein; 5 TA sites), which has no Tn insertions among the 14 independent libraries. Many of the ribosomal genes are categorized as essential or growth defect genes (15/23 *rpl* and 14/22 *rps* genes); most of those have sequences that were too short to analyze in previous studies. The essentiality of other small ORFs differs from conclusions of previous studies. For example, the *whiB1* transcription factor gene is classified as a growth defect gene, which is consistent with reports of the inability to delete it by homologous recombination (35–37), even though it was classified as nonessential in the initial TraSH study, likely due to the low resolution of that method (15).

10.1128/mBio.02133-16.2 FIG S2  Histogram of the sizes of essential genes identified in this study (blue bars) compared to the 2011 study by Griffin et al. (4) (red bars), showing that analysis with an HMM using more replicates enables detection of smaller essential genes. Download FIG S2, TIF file, 3.8 MB. Copyright © 2017 DeJesus et al. 2017 DeJesus et al. This content is distributed under the terms of the Creative Commons Attribution 4.0 International license.

To assess the relative impacts of both the high saturation of the libraries and the motif-dependent HMM on the detection of small ORFs, we compared our results to those produced by the Gumbel method, introduced by Griffin et al. (4), which uses the extreme-value distribution to identify unusually long stretches of sites without insertions (or “gaps”). To eliminate the growth condition (medium) as a variable, the Gumbel method was run on two of the libraries utilized in this study (grown in 7H9/10). The saturation of the combined data sets selected was 55.4%, which is similar to the saturation reported for previous TnSeq studies of H37Rv. The Gumbel method identified 0 small ORFs as essential, classifying all 1,274 as nonessential after correcting for multiple comparisons (the shortest gap that is significant at 55% saturation, based on the Gumbel distribution, is 10 TA sites long).

The inability of the Gumbel gap analysis method to identify small essential ORFs is primarily due to the saturation of the library. When all 14 libraries were analyzed instead, the method identified 93 small essential ORFs, and 84 of these (90.3%) are also identified as essential using the motif-dependent HMM. However, several of the remaining genes, which our motif-dependent HMM classifies differently, contain sites matching the nonpermissive motif. For instance, Rv3673c contains 3 empty sites that match the nonpermissive motif. While this can lead the method used by Griffin et al. (which focuses on identifying empty gaps) to identify the gap containing these sites as essential, the reduced probability of insertion at those sites makes it much likelier than they were missing by chance. Thus, taking this sequence-dependent bias into consideration can help better discriminate the essentiality for small ORFs.

### Association of essential regions with small RNAs.

We then assessed the association of unoccupied TA sites with sRNAs, which are short, highly structured transcripts that typically serve regulatory roles by modifying mRNA expression (38). sRNAs are often encoded as independent transcripts in intergenic regions but may also be derived from the 5′ untranslated region (5′UTR) or 3′UTR of an mRNA encoding a transcript (Storz et al. [39], Chao et al. [40], Loh et al. [41]). To date, these genomic features have not been included in TnSeq analyses because they contain only a few TA sites. In addition, there has been little consensus between studies describing the identification of sRNAs and their boundaries (42–45).

Determining the essentiality of the sRNAs first required definition of a set of sRNAs with accurate boundaries. We used a modified version of a bacterial small RNA sequencing (sRNA-Seq) protocol that utilizes size fractionation to enrich for small transcripts and ligate adapters to the natural ends of the transcripts (46). We next created a computational analysis, BS_finder (bacterial sRNA finder), to identify candidate sRNAs and distinguish them from other small transcript fragments such as those created through RNA degradation. BS_finder utilizes a sliding-window approach to identify small transcripts encoded completely or partially in an intergenic region with significantly greater read depth than the surrounding regions and sharp 5′ and 3′ boundaries. We employed stringent threshold criteria to identify 62 high-confidence sRNAs, ranging in size from 40 nucleotides (nt) to 268 nt, with an average size of 101 nt (Fig. S3a and Table S4). These sRNAs were named using the nomenclature proposed by Lamichhane et al. [47]. There was modest overlap between the sRNAs identified here and the sRNAs previously identified in studies enumerating intergenic transcripts or putative sRNAs (Fig. S3b) (Wang et al. [42], Arnvig et al. [48]). Many of the published putative sRNAs failed to reach our depth and boundary criteria designed to distinguish them from mRNA degradation products, but differences also likely reflect alterations in culture conditions, growth phases, and methods of library preparation. However, where the 5′ and 3′ boundaries of sRNAs have been experimentally determined by 5′ and 3′ rapid amplification of cDNA ends (RACE), there was excellent concordance with our analysis (45, 48–50). Of the 14 experimentally mapped *M. tuberculosis* sRNA ends, BS_finder mapped 12 within 3 bp of the experimentally defined end (Fig. S3c).

10.1128/mBio.02133-16.3 FIG S3  (a) Size distribution of high-confidence sRNAs found by BS_finder. (b) Venn diagram of the overlap of the sRNAs found by BS_finder, the sRNAs found by Wang et al., and the highly transcribed intergenic regions identified by Arnvig et al. (c) number of ends mapped at different distances from the published end. Download FIG S3, TIF file, 2.7 MB. Copyright © 2017 DeJesus et al. 2017 DeJesus et al. This content is distributed under the terms of the Creative Commons Attribution 4.0 International license.

10.1128/mBio.02133-16.7 TABLE S4  List of high-confidence sRNAs identified by the BS_finder method. Download TABLE S4, XLSX file, 0.01 MB. Copyright © 2017 DeJesus et al. 2017 DeJesus et al. This content is distributed under the terms of the Creative Commons Attribution 4.0 International license.

Using the HMM and our model (see Materials and Methods) to determine whether each sRNA is essential, 7 high-confidence sRNAs (ncRv0810c, ncRv0897, ncRv11315, ncRv1329, ncRv12783c, ncRv13418cA, and ncRv13418cB) and one previously identified sRNA, ncRNA3583A, were found to be essential or associated with a growth defect *in vitro*. Six of the essential sRNAs identified in this analysis appear to share transcriptional start sites (TSS) with essential or growth defect ORFs on the basis of TSS mapping data (51). It is possible that these regions lack insertions due to polar effects on expression of the neighboring essential gene. However, they could also represent distinct processed 5′UTRs of longer transcripts. Processing of 5′ and 3′ UTRs to generate independent *trans*-acting sRNAs has been previously described in several organisms (52, 53). Thus, the association of these RNAs with essential ORFs does not eliminate the possibility that each represents an independently essential element. For example, ncRv12783c shares a TSS with the Rv2783c ORF. Additionally, one of the 2 TA sites between the annotated 3′ end of ncRv12783c and the translational start of Rv2783c tolerated insertions, supporting the possibility that Rv2783c and ncRv12783c are independently essential RNA species. It is possible that other small transcripts within this list are essential but cannot be confidently identified as such because they have only a single TA site. This is exemplified by the 4.5S RNA, which has a single TA site that tolerated no insertions. The 4.5S RNA is the RNA component of the signal recognition particle (SRP), which is essential in *Escherichia coli* (54). Importantly, the 4.5S RNA shares a TSS with Rv3722c in *M. tuberculosis* (Shell et al. [51]), exhibiting a pattern similar to that seen with the essential sRNAs identified here.

### Association of unoccupied TA sites with other genomic features.

In addition to sRNAs, we examined the essentiality of other known genomic features, including tRNAs, rRNAs, and other structural RNAs; promoters; 5′UTRs; terminator sequences; and DNA methylation sites (defined in Materials and Methods; see Table S5 for a full list). Table 3 contains a summary of the number of features that were found to be essential for each given type. A total of 21 of the 35 tRNAs with at least 2 TA sites were classified as essential, as expected (even though many tRNAs are small and contain only 3 TA sites on average). Eight of them, including Rvnt04/GlyU, were labeled as nonessential. The apparent dispensability of Rvnt04/GlyU (Gly tRNA) is likely due to redundancy with other Gly tRNAs annotated in the *M. tuberculosis* genome (Rvnt27/GlyV, Rvnt32/GlyT). The rRNAs (23S, 16S, 5S*—rrs*, *rrl*, *rrf*), as well as *rnpB* (nucleic acid component of RNase P), were all classified as essential, though *ssr* (which binds to and regulates the RNA polymerase) was nonessential. Conversely, almost all of the rho-independent terminators were observed to be nonessential, suggesting a lack of essential functionality under these conditions. A subset of promoter regions (~4.0%) was found to be essential. Several of these promoters were upstream of ORFs that are themselves essential, such as Rv0440/GroEL and Rv0667/RpoB. However, the correlation of essential promoters to essential genes was not strong, which could have been due to several factors. In some cases, promoters were classified essential not because the gene that they regulated was essential but due to overlap of the coding region of an adjacent essential gene. More generally, the choice of a broad window (−150 to +70 bp) around the transcription start site may have led to indiscriminate inclusion of parts of the upstream sequence that did not affect gene expression.

10.1128/mBio.02133-16.8 TABLE S5  List of essentiality calls for genomic features in the *M. tuberculosis* H37Rv genome, obtained by using the motif-dependent hidden Markov model of essentiality. Final call: essential (ES), essential domain (ESD), growth defect (GD), nonessential (NE), growth advantage (GA), and uncertain (for short features). Download TABLE S5, XLSX file, 0.3 MB. Copyright © 2017 DeJesus et al. 2017 DeJesus et al. This content is distributed under the terms of the Creative Commons Attribution 4.0 International license.

**TABLE 3:** Essentiality of non-ORF genomic features

Feature	Total no.	No. with ≥2 TA sites	No. essential
sRNA	62	48	7
tRNA	45	35	21
rRNA and other structural RNAs	5	5	5
DNA methylation site	362	55	0
Predicted rho-independent terminator	148	73	2
5′UTR	1,558	1,003	39
Promoter region	2,060	1,841	57

### Identification of novel unannotated regions that are essential in the *M. tuberculosis* genome.

Aside from ORFs, RNAs, and other annotated features, we wished to use our saturated library to discover novel genomic regions that are essential for *in vitro* growth. We segmented the remaining unannotated regions of the genome (i.e., excluding ORFs, RNAs, and other annotated features) into contiguous regions that were labeled as belonging to the same state (as determined by the HMM). A total of 17 unannotated regions were identified as essential, and 12 were identified as associated with a growth defect (Table S6). These regions could represent functionally important features whose function is not yet known. Among the largest of the unannotated growth defect segments was an intergenic region between Rv3616c (*espA*, encoding ESX-1-associated protein) and Rv3617 (*ephA*, encoding epoxide hydrolase), spanning 15 TA sites. Another large segment spanning 13 sites occurred in the intergenic region between Rv1056 and Rv1057 (both hypothetical genes). Interestingly, while all four of these genes are nonessential, the intergenic regions contain multiple MprA binding sites (55, 56). MprAB is a two-component regulator implicated in stress response. Hence, we speculate that transposon insertion in the intergenic region might attenuate growth *in vitro* by disrupting the MprA regulon.

10.1128/mBio.02133-16.9 TABLE S6  List of unannotated essential regions in the *M. tuberculosis* H37Rv genome, obtained by using the motif-dependent hidden Markov model of essentiality. Final call: essential (ES), essential domain (ESD), growth defect (GD), nonessential (NE), growth advantage (GA), and uncertain (for short regions). Download TABLE S6, XLSX file, 0.1 MB. Copyright © 2017 DeJesus et al. 2017 DeJesus et al. This content is distributed under the terms of the Creative Commons Attribution 4.0 International license.

## DISCUSSION

This work describes the first use of a *Himar1* library (compiled from 14 independent replicates) that has reached the practical limit of saturation for the definition of essential genomic regions. The high level of saturation reduces the ambiguity associated with TA sites lacking insertions, allowing us to determine essentiality with high confidence for even small genomic features. In this context, we define an “essential” feature as one whose disruption causes complete absence or significant suppression of read counts among the combined data sets. Using a hidden Markov model to segment the genome into different regions of essentiality, we identified 625 ORFs that are essential for optimal growth *in vitro* (including domain essentials and growth defect genes). This set was largely consistent with previous studies (4), though there were some differences. Some of these discrepancies could be explained by differences in growth medium, since the use of 7H9/10 plus OADC provides carbon sources and metabolites lacking in the minimal medium used by Griffin et al. (4). However, most differences were due to the identification of smaller ORFs (with as few as 2 TA sites) than was possible in previous studies. In contrast, the Griffin study (4) was unable to categorize ORFs with fewer than 9 TA sites as “uncertain” (after correcting for multiple tests), due largely to the lower level of saturation. The density of mutagenesis achieved in this study was due to a combination of factors—both the large number of random mutants generated and the relatively small number of TA sites in the GC-rich *M. tuberculosis* genome. Equivalent saturation of less-G+C-rich organisms with a much larger number of TA sites would require a correspondingly larger number of independent mutants.

If *Himar1* insertion into TA sites were random, the analysis of this data set would have been straightforward, as it would be highly unlikely that even a single TA site in a nonessential region would be unrepresented in all 14 libraries. Finding that the apparent saturation of our library plateaued at less than 100% in otherwise well-represented (i.e., high-coverage) regions prompted us to investigate the sequence specificity of transposition. We identified a local sequence pattern, (GC)GNTANC(GC), associated with TA sites with few to no insertions, suggesting that these sites are nonpermissive for *Himar1* insertion. This effect was observed in *Himar1* TnSeq data sets from other organisms as well. This appears to be specific to *Himar1*, since other transposons such as Tn*5* do not appear to be inhibited from integrating at such sites. This putative nonpermissive sequence pattern is similar to the complement of a preference bias reported for insertion of the Sleeping Beauty transposon, ANNTANNT, into the human genome, based on a consensus of 138 unique insertion sites (57). Like *Himar1*, Sleeping Beauty is in the *mariner* class of transposons (58). Unlike Sleeping Beauty, we observed a sequence pattern only in our set of nonpermissive TA sites, while permissive sites (those with high insertion counts) showed no apparent sequence bias. Mechanistically, the sequence preference for *Himar1* could be due to effects of the G and C at ±2 bp on local DNA structure (59) or possibly the hydrogen bonding between the transposase and edges of nucleotides in the major groove of the DNA (60). An alternative explanation could be that accessibility for transposon insertion at certain TA sites might be blocked by the presence of a DNA-binding protein recognizing a specific binding-site motif. For example, it has recently been shown that the insertion-site specificity pattern for *Himar1* in *Vibrio cholerae* is influenced by the binding of histone-like nuclear structuring protein, *h-ns* (61). However, blocking by DNA-binding proteins seems unlikely, given that the Tn*5* transposon appears to integrate at sites matching the NP pattern with same frequency as at other sites.

The *Himar1* transposon is widely reported to have little sequence specificity, other than the requirement for TA dinucleotides. No other local sequence dependence has been observed to date, although a weak preference for more bendable regions of DNA in the *E. coli* genome has been previously reported (14). As a result, most previous gene essentiality studies using *Himar1* have been based on the assumption that insertion is equally probable at all TA sites. Our studies indicate that this assumption is not completely accurate and that insertions at TA sites matching the NP pattern are substantially less frequent than at other TA sites. In fact, the insertion bias of the *Himar1* transposon does not appear to be limited to mycobacteria, as it could be observed in TnSeq data sets of other organisms as well. This insertion bias could be a consequence of the transposase-DNA interaction, which involves significant distortion of the DNA, as observed in the recently described crystal structure of the transposase-DNA strand transfer complex (21), hence biasing against sequences that cannot accommodate this distortion. Because nearly 10% of all TA sites in the *M. tuberculosis* genome match the identified nonpermissive sequence pattern, indiscriminate inclusion of these nonpermissive sites in TnSeq analyses could artificially inflate the number of predicted essential regions determined by the use of a statistical framework that assumes random integration. Indeed, this insertional bias of *Himar1* may have contributed to the previous misclassification of genes in certain families, such as the MmpL and PE_PGRS genes. However, there is general agreement between our analysis of a saturated library and previous studies using subsaturated libraries (analyzed with statistical methods that assume unbiased random insertion). Thus, in subsaturated libraries, where nonpermissive sites represent a smaller subset of all sites lacking insertions, the assumption of random integration appears to have a less-significant impact on essentiality analyses. It is primarily in the context of nearly complete saturation that the insertional preferences of *Himar1* need to be taken into account.

The statistical analysis that we employed in this study was a hidden Markov model that takes advantage of both the identified sequence preference for insertion and the high level of saturation. Specifically, the HMM uses geometric distributions as likelihood functions to evaluate the read counts observed at TA sites in the entire genome, conditioning parameters based on whether the sites match the motif identified in this study. A significant advantage of the HMM over other analysis methods is that it is capable of determining different degrees of essentiality, representing increased or decreased levels of fitness. This includes growth defect genes, which exhibit an intermediate level of transposon insertions possibly reflecting a reduction in fitness, and growth advantage genes which result in an improvement in fitness when disrupted. Another advantage of the HMM is that the analysis is not limited to predefined gene boundaries but is instead capable of assessing essentiality across the entire genome in an unbiased (non-gene-centered) way. The high-resolution analysis afforded by the nearly complete saturation of the library enabled us, in an unprecedented way, to analyze the essentiality of smaller features of the *M. tuberculosis* genome, such as RNAs and regulatory regions such as promoters and terminators. Other than RNAs, the most frequent essential feature was the presence of 5′UTRs and promoter regions, while only a few of the predicted terminators and DNA methylation sites appeared to be essential. This could imply that adenosine methylation and rho-independent termination are not functionally relevant under these conditions, or possibly that the sequence models used to predict these sites are imperfect. We identified some essential regulatory regions (promoters and 5′ UTRs), though they were not well correlated with essential genes. We also developed a reliable list of 62 sRNAs based on RNA-Seq analysis, which took advantage of a customized sequencing protocol using direct adapter ligation to precisely characterize both 5′ and 3′ ends of the sRNA transcripts. Seven sRNAs were found to be essential for optimal growth *in vitro*, six of which occurred in 5′UTRs of essential genes and likely represent processed transcripts. Taking the data together, the results of high-resolution statistical analysis of essential regions of the genome, in conjunction with the high-resolution annotation of sRNA, will provide a useful reference for the *M. tuberculosis* research community. More broadly, these studies represent an experimental and statistical framework to extend saturation mutagenesis to other organisms.

## MATERIALS AND METHODS

### Construction of Tn libraries.

*M. tuberculosis* H37Rv transposon libraries were constructed by *Himar1* mutagenesis as previously described (17). Briefly, 100 ml of mid-log-phase *M. tuberculosis* culture (optical density at 600 nm [OD_600_], ~0.7 to 1.0) was incubated with 1 × 10^11^ to 2 × 10^11^ PFU of ΦMycoMarT7 phage (62) at 37°C for 4 to 18 h; cultures were then washed and plated on the media indicated in Table S1 in the supplemental material and incubated for 18 to 21 days at 37°C. Libraries with titers of greater than 100,000 CFU were used in the analysis to ensure comprehensive coverage of the possible TA dinucleotide insertion sites in the *M. tuberculosis* genome.

### Sequencing of Tn libraries.

Genomic DNA was extracted from the transposon libraries, and mutant composition was determined by sequencing amplicons of the transposon-genome junctions as previously described (17). Briefly, genomic DNA was sheared into ~500-bp fragments by ultrasonication by the use of a Covaris E220 system, and fragments were subjected to end repair and A-tailing with *Taq* polymerase and ligated to T-tailed adapters bearing random 7-nucleotide barcodes to distinguish between unique fragments before subsequent PCR amplification. Fragments containing transposon-genome junctions were selectively enriched in a first PCR amplification, size selected in the 400-to-600-bp range, and amplified in a second heminested PCR to add adapter sequences for Illumina sequencing. PCR amplicons were subjected to 75-to-100-bp paired-end sequencing on an Illumina HiSeq platform, and raw sequence data were exported to fastq files for further analysis.

### Data processing.

The sequence data were processed using the TPP tool included with TRANSIT (12). Reads were mapped to the genome using the Burroughs-Wheeler Aligner (bwa [63]). The set of reads in “read1” with a prefix matching the end of the *Himar1* transposon were mapped to the corresponding TA site in the genome (stripping off the transposon prefix). The read counts were reduced to template counts by discarding duplicates with the same barcode in “read2.” The final template counts were normalized across all the data sets using Trimmed Total Reads (TTR) normalization. TTR normalizes data sets so that they have the same mean template count, while ignoring (“trimming”) the top and bottom 5% of read counts to reduce the influence of outliers.

### RNA extraction, library construction, and sequencing.

*Mycobacterium tuberculosis* H37Rv was cultured in 7H9 media (Difco Laboratories) with 10% oleic acid-albumin-dextrose-catalase, and cells were harvested at an OD of 1.0. Cell pellets were resuspended in TRIzol reagent (Ambion, Life Technologies, Inc.) and lysed using a FastPrep 24 instrument. RNA was purified using a Direct-Zol MiniPrep kit (Zymo Research), and DNA was digested using Turbo DNase (Ambion, Life Technologies, Inc.). Size selection to achieve an approximately 300-nt cutoff was performed using RNA Clean and Concentrator-25 columns (Zymo Research) according to the manufacturer’s instructions with the following modification: preparation of the adjusted RNA binding buffer was done by adding an equal volume of RNA binding buffer and 70% ethanol. The small RNA fraction was then depleted of rRNA using a RiboZero-Bacteria kit (Illumina). The small RNA library was then prepared as described previously using a modified TruSeq small-RNA-sequencing kit (Illumina). Briefly, RNA was dephosphorylated using RppH enzyme (New England BioLabs) followed by an ethanol precipitation step. 3′ and 5′ adapters were then ligated to the RNA followed by reverse transcription and PCR amplification. The libraries were then purified using Agencourt AMPure XP beads (Beckman Coulter, Inc.). Paired-end sequencing (150 bp) was then performed on a MiSeq sequencer. Sequenced reads were aligned to the *Mycobacterium tuberculosis* NC_000962.3 genome (National Center for Biotechnology Information database) using the Burrows-Wheeler alignment tool (63).

### sRNA identification.

Per-base coverage of the genome was obtained using the *genomecov* tool of the BEDtools suite (64). sRNAs were then identified using BS_finder (bacterial sRNA finder), which uses a sliding-window approach to sRNA identification. Briefly, a sliding window scans the per-base read depth and searches for positive slope (the intensity of which can be modified to adjust stringency) across the window, which demarcates a 5′ end (searching the plus strand) or a 3′ end (searching the minus strand) if the position passes a modifiable read depth threshold. A nested sliding window then searches for decreases in slope across the window, which demarcates either a 3′ or 5′ end. If the discovered feature is not entirely within an ORF and has a 5′ end at least 50 bp away from the nearest ORF translational start site, then it is output as a candidate sRNA. BS_finder was run on the data set using default parameters: a sliding-window size of 2 bp with a slope threshold of 3 and a read depth threshold of 500.

### Statistical analysis of essentiality.

The hidden Markov model described in reference 25 was extended to take into consideration the suppression of insertions observed at sites matching the NP motif. Read counts were modeled as coming from four different states of essentiality: the essential, growth defect, nonessential, and growth advantage states. Geometric distributions, conditioned on the local sequence (i.e., matching the NP motif or not), were used to assess the likelihood of observing a read count from a given state as follows:

$$P\;(O_{t}|Q_{t}=s,\;np=0)=\text{geometric}\left(O_{t}|P=\frac{1}{\mu_{s}}\right)$$

$$P\;(O_{t}|Q_{t}=s,\;np=1)=\text{geometric}\left(O_{t}|P=\frac{1}{\mu_{s}\times \omega }\right)$$

where *O_t_* values represent the insertion counts observed at each site (normalized and summed over the 14 replicates), *Q_t_* represents the state labels (*Q_t_* ∈ <ES, GD, NE, GA>), μ_*s*_ represents the expected read count for state *s* (which is chosen to represent different levels of fitness [25]), *np* indicates whether the site matches the NP motif (1) or not (0), and ω represents the ratio between the mean read count at sites matching the NP motif and the mean read count at other sites (accounting for the reduction in read counts at nonpermissive sites) as follows:

$$\omega =\frac{\mu_{np}}{\mu_{P}}$$

The transition probabilities were set such that the states would have a high probability of remaining in the current state, thus requiring a significant change in local read counts to prompt a change of the essentiality state (25). The Viterbi algorithm (65) was used to identify the most likely state sequence for the entire genome.

To obtain essentiality classifications for individual ORFs or features, we employed the following criteria. Most of the ORFs were classified by the plurality of state labels (the most frequent) among its TA sites. ORFs which have an essential domain (i.e., a significantly large ES segment, as well as a nonessential region spanning at least 200 bp) were identified using the extreme-value distribution by calculating the probability of observing the corresponding sequence of ES states in a row using the formulas given in reference 4. Small ORFs (≤3 TA sites), which could be influenced strongly by the essentiality of surrounding sites, were classified as essential only if they were significantly devoid of insertions as determined by a binomial distribution [*P* value < 0.05; $P\;(k;\;n,\;P)=\sum_{i=0}^{k}\left(in\right)\;P^{i}\;{\left(1\;-\;P\right)}^{n-i}$], provided that they span at least 15 bp. Small ORFs which did not pass this threshold but which were completely devoid of insertions nonetheless (mostly ORFs with just 1 TA site) were considered to be “uncertain.”

### Analysis of non-ORF genomic features.

Promoter regions were defined based on a set of 2,060 transcriptional start sites (TSSs) defined in reference 51 that are within 500 bp of a translational start site (on the same strand). A region around each TSS (−150 to +70 bp) was used to define the promoter region, since regulatory signals such as transcription factor binding sites are often found in this broad region (not just the −10 and −35 regions of the sigma factor binding site) (66). 5′UTRs were defined as the region between the transcriptional and the translational start sites (for transcripts with leaders), which can contain regulatory features such as ribosomal binding sites or riboswitches. The motif “GATN_4_RTAC” was used to define DNA methylation sites, since it is the only 1 of 3 motifs identified to date (27, 67) that has the possibility of containing a TA site and thus of being capable of being disrupted by a *Himar1* transposon insertion. Finally, a predictive model for rho-independent termination signals (~65-bp pseudopalindromic sequence) was applied to identify putative terminators in the 3′ ends of transcripts (68).

The essentiality of these features, together with the remaining unannotated regions in the genome, was analyzed using the method outlined above.

### Accession number(s).

All TnSeq data sets are publicly available on the NCBI Sequence Read Archive with accession number SRP083947 and BioProject accession number PRJNA341349.
