# Supplementary material for: Comprehensive Essentiality Analysis of the Mycobacterium tuberculosis Genome via Saturating Transposon Mutagenesis
Source: mBio. 2017 Jan 17;8(1):e02133-16. doi: 10.1128/mBio.02133-16 (PMC5241402; doi:10.1128/mBio.02133-16)
Supplement: TABLE S1 [file mbo002173137st1.docx]

| **Library** | **Medium** | **Total Reads** | **Mapped Templates** | **#TA sites hit** | **% coverage** |
| --- | --- | --- | --- | --- | --- |
| WX-WT1 | 7H9 agar, Tw, Kan | 6,418,175 | 995,189 | 47,768 | 64 |
| WX-WT2 | 7H9 agar, Tw, Kan | 4,691,828 | 888,503 | 49,309 | 66.1 |
| WX-WT3 | 7H9 agar, Tw, Kan | 5,319,709 | 3,195,442 | 39,441 | 52.9 |
| WX-WT4 | 7H9 agar, Tw, Kan | 5,127,111 | 3,042,234 | 46,408 | 62.2 |
| SWP-WT1 | 7H10, Tw, Kan | 2,616,758 | 1,127,848 | 34,843 | 46.7 |
| SWP-WT2 | 7H10, Tw, Kan | 3,993,696 | 1,764,177 | 37,965 | 50.9 |
| SWP-WT3 | 7H10, Tw, Kan | 1,635,840 | 1,022,842 | 39,606 | 53.1 |
| SWP-WT4 | 7H10, Tw, Kan | 5,180,853 | 3,155,939 | 46,025 | 61.7 |
| SWP-WT5 | 7H10, Tw, Kan | 3,150,964 | 1,150,053 | 31,464 | 42.2 |
| SWP-WT6 | 7H10, Tw, Kan | 3,832,963 | 1,590,073 | 38,156 | 51.1 |
| SWP-WT7 | 7H10, Tw, Kan | 9,463,790 | 4,093,724 | 40,452 | 54.2 |
| CB-WT | 7H10, Tw, Kan | 15,902,338 | 7,798,985 | 47,008 | 63 |
| TraCS-053 | 7H10, Tw, Kan | 7452993 | 3,432,553 | 37,201 | 50 |
| TraCS-054 | 7H10, Tw, Kan | 7271941 | 2,056,856 | 39,689 | 53.2 |
