# Supplementary figures and images for: Comprehensive Essentiality Analysis of the Mycobacterium tuberculosis Genome via Saturating Transposon Mutagenesis
Source: mBio. 2017 Jan 17;8(1):e02133-16. doi: 10.1128/mBio.02133-16 (PMC5241402; doi:10.1128/mBio.02133-16)

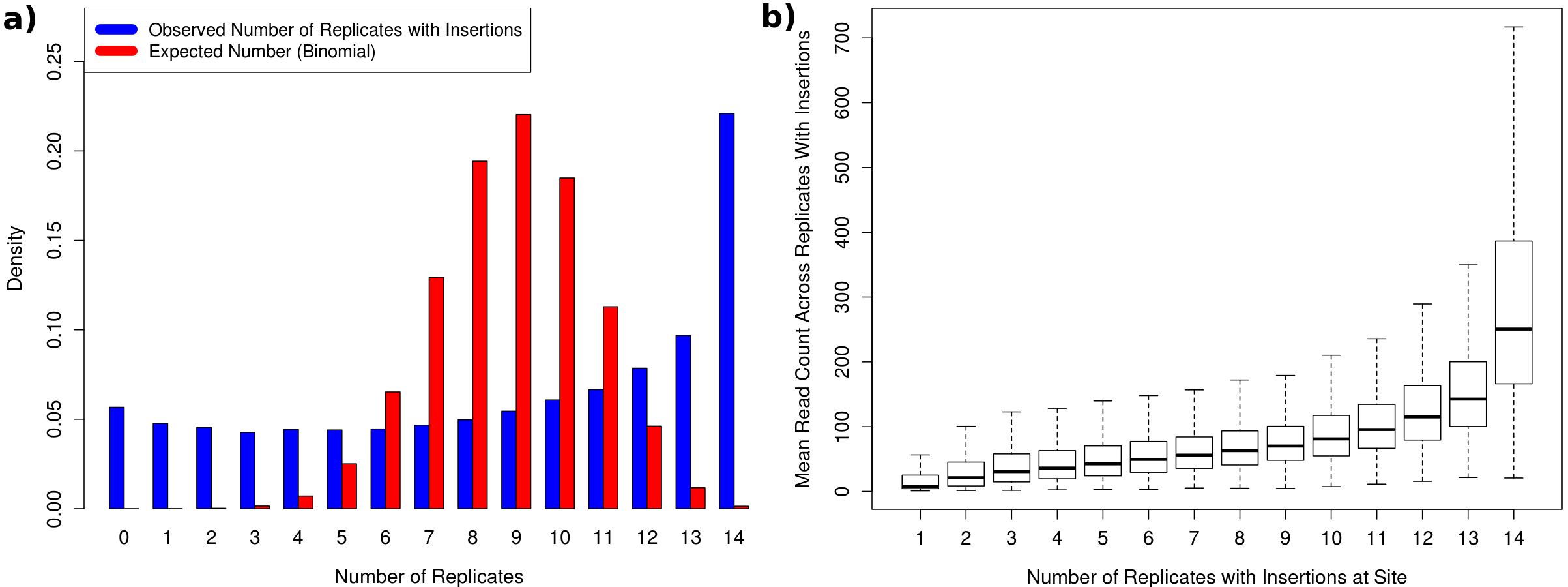

Supplement: FIG S1 [file mbo002173137sf1.tif]

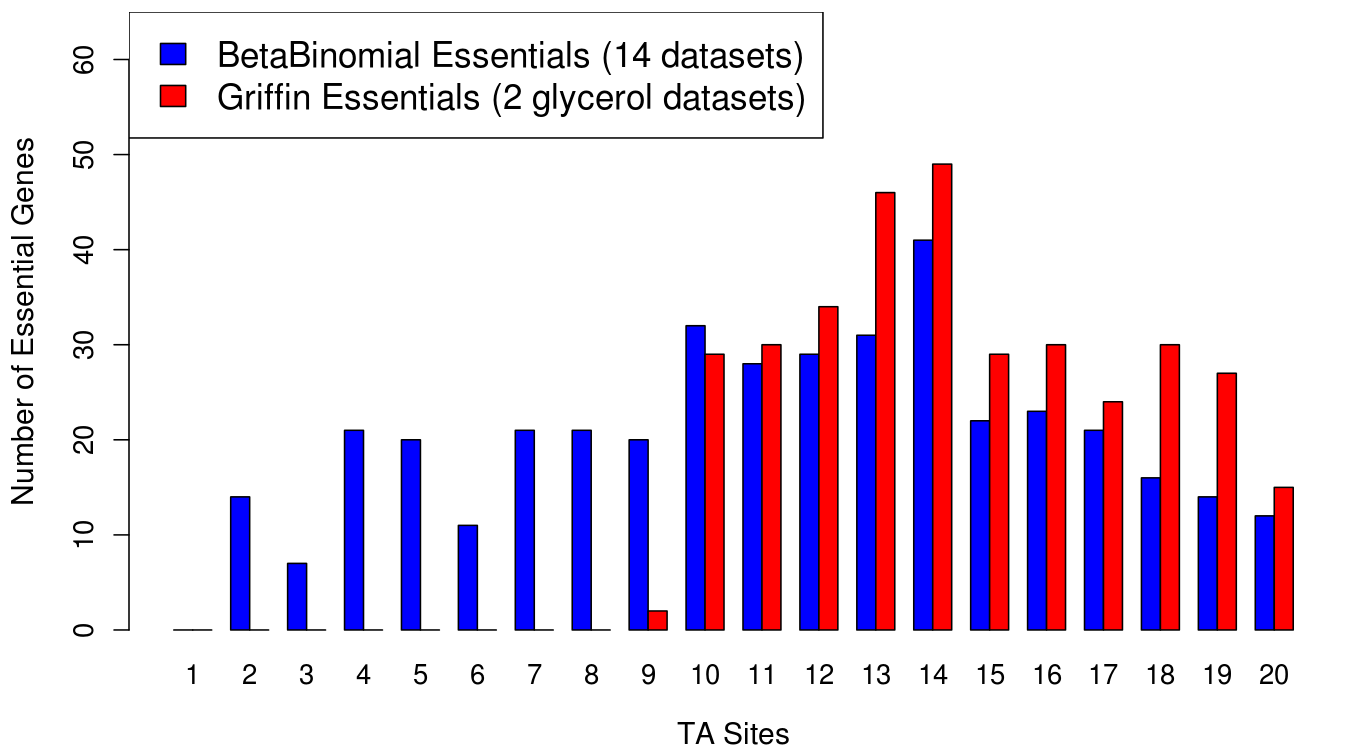

Supplement: FIG S2 [file mbo002173137sf2.tif]

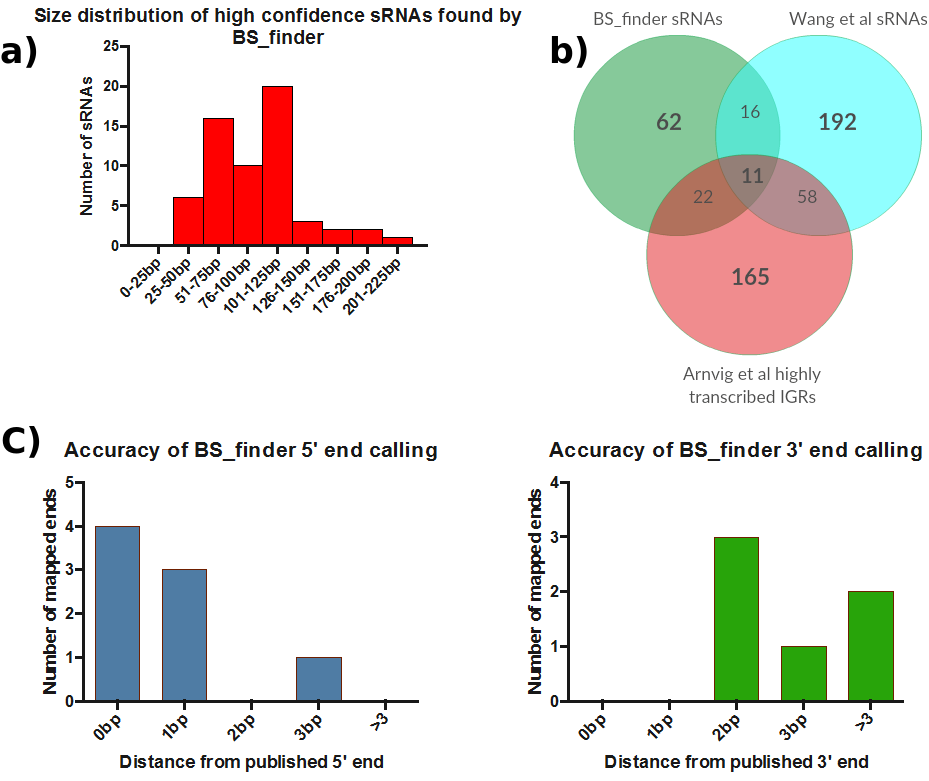

Supplement: FIG S3 [file mbo002173137sf3.tif]
