# Supplementary material for: Comprehensive Essentiality Analysis of the Mycobacterium tuberculosis Genome via Saturating Transposon Mutagenesis
Source: mBio. 2017 Jan 17;8(1):e02133-16. doi: 10.1128/mBio.02133-16 (PMC5241402; doi:10.1128/mBio.02133-16)
Supplement: TEXT S1 [file mbo002173137s1.pdf]

Supplementary Material for "Comprehensive essentiality analysis of the *Mycobacterium tuberculosis* genome via saturating transposon mutagenesis"

Michael A. DeJesus, Elias R. Gerrick, Weizhen Xu, Sae Woong Park,
Jarukit E. Long, Cara C. Boutte, Eric J. Rubin, Dirk Schnappinger,
Sabine Ehrt, Sarah M. Fortune, Christopher M. Sassetti, and Thomas R. Ioerger

November 29, 2016

Segmentation algorithm for identifying low-coverage regions

In order to identify low-coverage (LC) regions in the genome (i.e. regions associated with smaller counts and less insertion frequency than average), we implemented a Hidden Markov Model (HMM) with two states: LC (for low-coverage regions), and HC (for high-coverage regions). The data for the HMM was the mean read-count over the 14 datasets, for each site in the genome. The emission probabilities for the states were modeled as geometric distributions:

$$p(O_i \mid S_i = LC) = \text{Geometric}(O_i \mid p = 0.1075)$$
$$p(O_i \mid S_i = HC) = \text{Geometric}(O_i \mid p = 0.0107)$$

with parameters that correspond to an expectation that observations at LC sites will have a 10-fold reduction in the mean read-count, while observations at HC sites will have read counts similar to the mean read-count of the library. Transition probabilities of the HMM were set to have a low probability of remaining in the same state, thus requiring the data at the sites to be highly consistent with the likelihood in order to remain in a state:

$$A = \begin{vmatrix} 0.01 & 0.99 \\ 0.99 & 0.01 \end{vmatrix}$$

This prevents rapidly switching between states. Finally, the state calls were obtained using the Viterbi algorithm, which determines the most likely sequence of states that would produce the observed sequence of observations.
